# A Comprehensive Neurological Study on the Relationship Between Smoking Cigarettes and Alzheimer’s Disease: Potential Effects of Transitioning to E‐Cigarettes

**DOI:** 10.1002/alz.087309

**Published:** 2025-01-09

**Authors:** Youssef A. Ismail, Youssef M. Abd El‐Satar, Youssef Haitham, Hazim Mohamed, Mohammad Walid

**Affiliations:** ^1^ Faculty of Medicine Port Said Univeristy, Egypt, Port Said, Port Said Egypt; ^2^ STEM Neurology & Neuropsychological0 Research Group Egypt (SNRGE), Port Said, Port Said Egypt; ^3^ Faculty of Medicine Cairo Univeristy, Egypt, Cairo, Cairo Egypt; ^4^ Faculty of Medicine Suez Canal Univeristy, Egypt, Ismailia, Ismailia Egypt; ^5^ Faculty of Medicine Helwan University, Cairo, Helwan Egypt; ^6^ Faculty of Medicine, Arish University, Arish, North Sinai Egypt

## Abstract

**Background:**

Undergoing intense research and investigation, smoking has garnered attention due to its extensive implications for public health, particularly focusing on cardiovascular and pulmonary issues. however In comparison, the neurological effects of nicotine have not yet been thoroughly investigated. Our objective is to investigate the relationship between smoking cigarettes and Alzheimer disease (AD) and possible effects of switching to E‐cigarettes.

**Methods:**

We reviewed database searches through October 2023 in MEDLINE, PsycINFO, and the Cochrane Central Register of Controlled Trials; and additional searches for ongoing trials through ClinicalTrials.gov, World Health Organization International Clinical Trials Registry Platform, and Current Controlled Trials (ISRCTN Register).

**Results:**

Nicotine has been observed to yield cognitive benefits and alleviate cognitive deficits associated with neurological diseases, such as Alzheimer’s disease (AD), in certain studies. Conversely, a notable increase in the risk of AD has been linked to active smoking in other studies, while another investigation suggests no discernible distinction between smokers and non‐smokers in this context. Although e‐cigarettes generally entail fewer adverse health effects, the neurological impact persists, attributed primarily to nicotine. As of July 2023, approximately 1.3 billion individuals, constituting approximately 16.2% of the global population, are smokers according to the World Health Organization (WHO). In the United States, 17.2% of adults are active smokers, with 11.1% employing traditional smoking methods. Notably, there is an observable decline in cigarette smoking, decreasing from 14.4% in 2019 to 11.1% in 2023, while the use of E‐cigarettes among US adults has risen from 4.4% to 6.1%. This trend is even more pronounced among youth, with around 14.1% of high school students and 3.3% of middle school students reporting e‐cigarette usage.

**Conclusion:**

The divergent findings regarding the impact of nicotine on cognition suggest the existence of an overlooked co‐factor associated with the effects of nicotine on the brain, warranting further investigation. Nevertheless, our analysis indicates that the shift to e‐cigarettes potentially elevates the risk from a neurological standpoint, as evidenced by documented cases of nicotinic overdosing, a phenomenon not possible with traditional cigarettes.

